# Analysis of the Damage Mechanism Related to CO_2_ Laser Cochleostomy on Guinea Pig Cochlea

**DOI:** 10.1155/2016/5982397

**Published:** 2016-12-14

**Authors:** Xiang Liu, Xiao-qing Qian, Rui Ma, Fang-Lu Chi, Dong-Dong Ren

**Affiliations:** ^1^Department of Otology & Skull Base Surgery, EYE & ENT Hospital of Fudan University, Shanghai 200031, China; ^2^Shanghai Clinical Medical Center of Hearing Medicine, Shanghai 200031, China; ^3^Key Laboratory of Hearing Medicine, Ministry of Health, Shanghai 200031, China; ^4^Department of Research Center, EYE & ENT Hospital of Fudan University, Shanghai 200031, China

## Abstract

Different types of lasers have been used in inner ear surgery. Therefore, it is of the utmost importance to avoid damage to the inner ear (e.g., hyperthermia and acoustic effects) caused by the use of such lasers. The aim of this study was to use a high powered fibre-enabled CO_2_ laser (10 W, 606 J/cm^2^) to perform cochleostomies on guinea pig cochlea and to investigate the possible laser-induced damage mechanisms. The temperature changes in the round window membrane, auditory evoked brainstem response, and morphological of the hair cells were measured and recorded before and after laser application. All of the outcomes differed in comparison with the control group. A rise in temperature and subsequent increased hearing loss were observed in animals that underwent surgery with a 10 W CO_2_ laser. These findings correlated with increased injury to the cochlear ultrastructure and a higher positive expression of E-cadherin and *β*-catenin in the damaged organ of Corti. We assume that enhanced cell-cell adhesion and the activated *β*-catenin-related canonical Wnt-signalling pathway may play a role in the protection of the cochlea to prevent further damage.

## 1. Introduction

The instruments used in surgical techniques for inner ear surgeries, such as stapedotomy and cochlear implantation, must be powerful enough to be efficient while, at the same time, preventing damage to the inner ear. Lasers with varying wavelengths, set to appropriate intensities, can fulfil two basic principal requirements: ablation of bone at a precise location and achievement of an ideal setting without penetrating deeply into the perilymph and causing injury to the sensory structures of the inner ear. The first laser stapedotomy was performed by Perkins in 1979 using an argon laser. Since then, different types of lasers, such as the erbium: yttrium aluminium garnet laser, potassium titanyl phosphate (KTP) laser, and CO_2_ laser, have been used in inner ear surgery [1]. The use of different lasers has been described in both experimental and clinical studies [2–8].

However, each laser has advantages and disadvantages. The application of a laser to any medium leads to absorptive, reflective, thermal, and acoustic effects. Visible lasers (argon and KTP) have a short wavelength (0.5 *μ*m) that can only be partially absorbed by the stapes footplate or the bone wall of the cochlea and readily passes through the perilymph to be absorbed by the tissues of the inner ear, leading to tissue damage [2]. The long wavelength of the invisible CO_2_ laser (10.6 *μ*m) is well-absorbed by water. Absorption results in the conversion of laser energy into heat [7]. In our previous study, positive expressions of iNOS and Hsp70 were observed in spiral ganglion cells, nerve fibres, supporting cells of the organ of Corti, and cells of the spiral ligament after serious irradiation injury of the cochlear ultrastructure [5]. Therefore, the degree of temperature increase and which molecules are affected by the application of a high-intensity CO_2_ laser in a guinea pig cochlea model require further investigation.

This study aimed to explore how the thermal effect of lasers interferes with molecules in the damaged inner ear. Guinea pigs were chosen as the animal model because the basal turn of the cochlea is easily accessible and the thickness of the cochlea wall (1 mm anterior to a round window of approximately 120–160 *μ*m) is comparable to the dimensions of a slightly thickened otosclerotic footplate (150–200 *μ*m) [3]. We used a new type of handheld fibre-enabled CO_2_ laser, the 10 W (606 J/cm^2^) CO_2_ laser, to explore possible damage mechanisms in the inner ear.

## 2. Materials and Methods

### 2.1. Animals

Adult male albino guinea pigs (weight, 250–350 g) were used in this study. All 21 animals were obtained from the Animal Center of the Medical School of Fudan University and were housed under clear conditions. The Animal Research Control Committees of Fudan University approved the experimental protocols and procedures performed in this study.

An otoscopic examination was performed on all guinea pigs prior to surgery to ensure that the external auditory canals and tympanic membranes were normal. All animals had normal hearing with a positive Preyer reflex. Before each procedure, the guinea pigs were anaesthetised using ketamine (85 mg/kg body weight) and xylazine (7.5 mg/kg body weight). The left ear of each animal was chosen to be the experimental ear, and it received irradiation by a 10 W CO_2_ laser. Each animal's right ear was classed as the control group and was irradiated on the temporal muscle close to the cochlea.

### 2.2. Laser Device

Perforation of the cochlear bone was performed with the new AcuPulse 40 WG CO_2_ laser using the FiberLase flexible CO_2_ laser waveguide (Lumenis, Santa Clara, CA, USA). This laser has a handheld energy delivery system with a flexible 2 m long waveguide and a variety of rigid and malleable microsurgical accessories with different lengths and flexibilities.

### 2.3. Surgery

After the induction of anaesthesia, we created a postauricular incision in the left ear, exposed the bulla, fractured and removed the bony shell of the bulla, and carefully exposed the basal turn of the cochlea under a microscope (OPMI 9-FC, Carl Zeiss, Jena, Germany). The left ear of each guinea pig underwent perforation of the cochlea with the laser 1 mm anterior to the round window niche. The 10 W SuperPulse laser mode was used at a working distance of up to 3 mm in noncontact mode. Two exposure times were selected. Each laser shot continued for 0.1 s and the time interval between each shot was 0.1 s (Table 1). Cochleostomy was recognised by an effusion of perilymph. An opening of approximately 0.5 mm in diameter was obtained. The control group underwent the same procedure but were irradiated by equal lasers on the temporal muscles to exclude the influence of noise caused by the application of the laser. The guinea pig cochleae with their bony coverage were photographed with the help of a digital camera coupled to a microscope (OPMI 9-FC, Carl Zeiss, Germany).

### 2.4. Temperature Measurement

Temperature changes were measured with a type K (chromium and aluminium) thermocouple (TM6801, Jingda, China). The detection procedure was well documented in our previous article [6]. A 0.8 mm diameter detecting head was placed onto the membrane of the round window to measure the temperature change at approximately one pulse per second during laser irradiation in vivo. The temperature was recorded manually by a digital thermometer.

### 2.5. Measurement of the Auditory Brainstem Response

Recordings of the auditory brainstem response (ABR) were performed before and immediately after laser irradiation. Animals were anaesthetised and then placed in a sound-isolated and electrically-shielded booth (Ningbo, Tonecon Acoustic Systems, Nanjing, China). Acoustic stimuli were delivered monaurally via an earphone (Bio-logic Systems Corp., Mundelein, IL, USA) attached to a customised plastic speculum inserted into the ear canal. Subdermal electrodes were inserted at the vertex of the skull, under the right (ground) and left ear. The Bio-Logic NAVPR2 hearing diagnostic system (Natus Medical Inc., San Carlos, CA, USA) was used to provide the stimuli (click stimuli; duration, 0.1 ms) and record the response. Up to 1,024 responses were averaged for each stimulus level. ABRs were determined by reducing the intensity in 10 dB increments and then in 5 dB steps close to the threshold until no organised responses were detected. Thresholds were estimated at the lowest stimulus level at which a response was observed, identified by the presence of recognisable and repeatable wave IIIs. All ABR measurements were evaluated by an expert blinded to the treatment conditions.

### 2.6. Scanning Electron Microscopy

Three guinea pigs from each group were sacrificed under deep anaesthesia after postoperative ABR evaluation. The tympanic bullae opened and the bony coverage of the cochlea was removed. The cochleae were fixed in a 2.5% solution of glutaraldehyde in a 0.1 mol/L sodium cacodylate buffer. After fixation, the dissected cochleae were dehydrated in 10%, 30%, 70%, 90%, and 100% (3x) concentrations of ethanol. A critical point dryer (EM CPD300, Leica, Wetzlar, Germany) was used to dry the specimens with liquid carbon dioxide and the specimens were then sputter-coated with platinum-palladium (E-1045 ion sputter, Hitachi High Technologies Co., Tokyo, Japan). The surfaces of the organs of Corti were observed under a low-vacuum scanning electron microscope (SEM) (Nova NanoSEM 230, FEI Company, Hillsboro, OR, USA).

### 2.7. Immunofluorescent Staining

After the postoperative ABR evaluation, three additional guinea pigs from each group were sacrificed under deep anaesthesia for immunofluorescent staining. As outlined above, the tympanic bullae were removed and fixed in 4% paraformaldehyde in a phosphate-buffered saline solution (PBS) for a period of 24 h. Under magnification, the bony wall of the cochlea was removed with a pick and forceps to obtain the basilar membrane of the cochlea. The following staining procedure was performed as described previously [9]. The specimens were treated with 0.1% Triton X-100 plus 10% donkey serum for 30 min. They were then incubated overnight with primary antibodies at 4°C. The primary antibodies used were as follows: E-cadherin (1 : 200; BD Biosciences, Sparks, MD, USA) and *β*-catenin, (1 : 200; Santa Cruz Biotechnology, Inc., CA, USA). The preparation was rinsed 3–5 times in PBS and then incubated in the dark with secondary antibodies for 2 h at 37°C. The secondary antibodies used included donkey anti-mouse/rabbit Alexa Fluor 555 and/or donkey anti-mouse/rabbit/goat (H+L) Alexa Fluor 647 (1 : 1,000; Molecular Probes, Invitrogen Life Technologies, Carlsbad, CA, USA), and phalloidin (1 : 1000; Invitrogen). All specimens were examined with a Leica confocal laser-scanning microscope (Leica SP5, Leica Microsystems) and images were captured by the microscope.

### 2.8. Western Blot

Fifteen guinea pigs were sacrificed and separated into experimental and control groups. The basilar membranes were collected, as noted above, and lysed in a radioimmunoprecipitation assay buffer (50 mM Tris-HCl, 1% NP-40, 0.25% Na-deoxycholate, 150 mM NaCl, 1 mM Na_3_VO_4_ and NaF) containing protease inhibitors (1 *μ*g/mL each of aprotinin, leupeptin, pepstatin, ethylenediaminetetraacetic acid, and phenyl methyl sulfonyl fluoride) in microcentrifuge tubes and were sonicated for 10 s. They were then centrifuged at 12,000*g* for 15 min at 4°C. The supernatant was transferred to a new microfuge tube, mixed with a sample buffer (12 mM Tris-HCl, 96 mM glycine, 10% SDS, 1% 2-mercaptoethanol, and 0.1% bromophenol blue, pH 6.8), and boiled for 5 min. The total protein was determined by the bicinchoninic acid assay protein assay (Beyotime, Beyotime Institute of Biotechnology, Jiangsu, China), and aliquots of 40 *μ*g protein/lane for each sample were separated by electrophoresis in an 8% sodium dodecyl sulphate polyacrylamide gel using a 5% stacking gel. Resolved proteins were transferred to a nitrocellulose membrane and saturated for 30 min at room temperature with a blocking buffer (25 mM Tris (pH 8.0), 125 mM NaCl, 0.1% Tween 20, and 4% skim milk) and incubated with anti-E-cadherin (1 : 2500; BD Biosciences), anti-*β*-catenin (1 : 500; Santa Cruz Biotechnology), and anti-GAPDH (glyceraldehyde-3-phosphate dehydrogenase) (1 : 1000; Beyotime) antibodies for 1 h at room temperature and then overnight at 4°C, followed by incubation for 1 h in appropriate secondary antibodies conjugated with horseradish peroxidase (IgG-HRP; 1 : 1000; Beyotime, Beyotime). Immunoreactive bands were detected using an enhanced chemiluminescence system (Beyotime). Detection was performed by a Kodak imaging station 4000 MM Pro (Eastman Kodak, Rochester, NY, USA) and quantified using the ImageJ software.

### 2.9. Statistical Analysis

All statistical comparisons were performed with Student's *t*-test. Data analysis was undertaken using the SPSS software package (ver. 15.0; SPSS Inc., Chicago, IL, USA). A *P* value < 0.05 was taken to indicate statistical significance.

## 3. Results

### 3.1. Cochleostomy on Guinea Pig Cochlea

**Figure 1:**
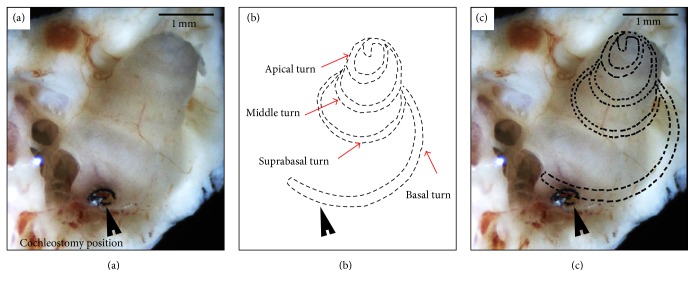
Cochleostomy was performed in the basal turn near the round window. (a) The perforation as the black arrow showed the cochleostomy position. (b) The cochlear membrane was delineated with dotted lines. The red arrows located the specimens from the basal turn, suprabasal turn, middle turn, and apical turn. (c) The site of the simulated cochlear membrane under the bone. Scale bar: 1 mm.

In this experiment, cochleostomy was performed in the basal turn near the round window. One cochlea of guinea pig with its bony coverage was shown in Figure 1. The perforation was clear and the membrane was delineated with dotted lines. The specimens from the basal membrane, suprabasal membrane, middle membrane, and apical membrane marked as the red arrows were collected and processed.

### 3.2. The Thermal Effect Was Intense in Animals Treated with a 10 W CO_2_ Laser

**Figure 2:**
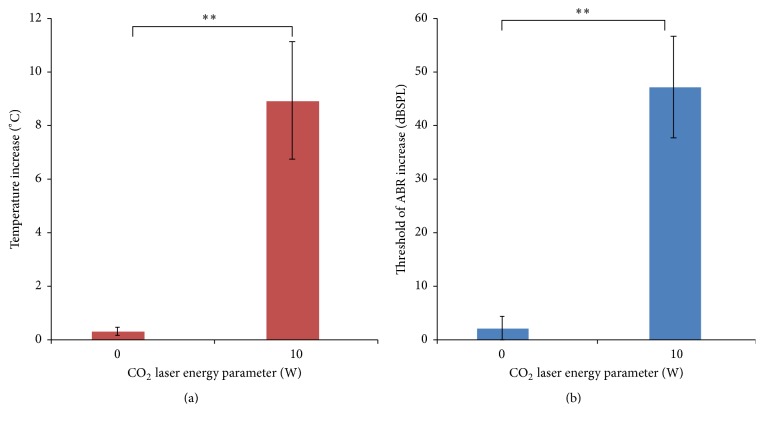
The temperature increase during surgery and mean incremental rise in the ABR threshold immediately after surgery by CO_2_ laser irradiation (10 W laser and control groups). (a) The temperature rise in the 10 W CO_2_ laser group was much higher than in the control group (*P* < 0.01). Data are presented as means + standard deviation (SD), *n* = 21, ^*∗∗*^ *P* < 0.01. (b) ABR threshold rise immediately after laser irradiation was higher in the 10 W laser group than the 0 W laser group (*P* < 0.01). Data are presented as means + SD, *n* = 21, ^*∗∗*^ *P* < 0.01.

Temperature changes were measured when applying the CO_2_ laser. As shown in Figure 2(a), the temperature increase was significantly higher in the 10 W CO_2_ laser group than in the control group (8.92 ± 2.13°C and 1.13 ± 0.32°C, respectively; *P* < 0.01).

### 3.3. Animals Had a Great Loss of Hearing When Treated with a 10 W CO_2_ Laser

The ABR thresholds were recorded before and immediately after surgery. The mean rise in the ABR threshold after surgery was calculated and is shown in Figure 2(b). A higher ABR threshold equates to a greater degree of hearing loss. The average ABR threshold increase in the two groups was 2.5 ± 2.65 dB SPL (sound pressure level; control group) and 47.5 ± 10.5 dB SPL (10 W laser group). After surgery, when compared with the control group, the animals in the 10 W CO_2_ laser group displayed a significantly large increase in their ABR threshold (*P* < 0.01).

### 3.4. Outer Hair Cell Collapse in Animals Treated with a 10 W CO_2_ Laser

**Figure 3:**
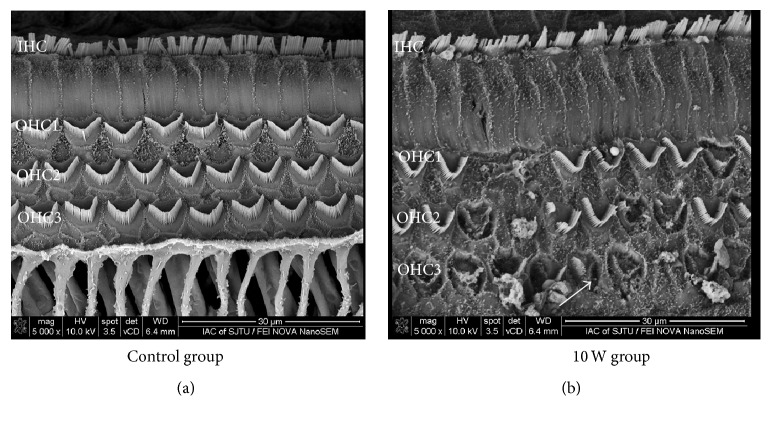
SEM image of the basal turn of a cochlea immediately after applying CO_2_ laser irradiation. (a) Image of the basal turn in the control group. The stereocilia and cuticular plates of the inner and outer hair cells and supporting cells showed a normal configuration. OHC1: first line of the outer hair cells; OHC2: second line of the outer hair cells; OHC3: third line of the outer hair cells; IHC: inner hair cells. Scale bar: 30 *μ*m. (b) Image of the basal turn after applying 10 W CO_2_ laser irradiation. Most of the outer hair cells had collapsed and derangements of the stereocilia were observed. Parts of the cells were missing, particularly in the third row of the outer hair cells (OHC3) in the basal turn (arrow), but the inner hair cells showed a normal configuration. Scale bar: 30 *μ*m.

After surgery, the cochleae of each group were collected and processed for examination under SEM. Images of the basal turn of animals treated with a 10 W CO_2_ laser are shown in Figure 3. The stereocilia and cuticular plates of the inner and outer hair cells and supporting cells showed a normal configuration in specimens in the control group (Figure 3(a)). In contrast, most of the outer hair cells had collapsed and derangements of the stereocilia were observed in the 10 W CO_2_ laser group (Figure 3(b)) with part of them missing, especially those in the third row of the outer hair cells (arrow) in the basal turn. However, the inner hair cells showed a normal configuration. All ultrastructure in other turns showed normal appearance as the control (Data not shown).

### 3.5. Cell-Cell Adhesion Was Enhanced and the *β*-Catenin-Related Canonical Wnt-Signalling Pathway Was Activated following 10 W CO_2_ Laser Treatment

**Figure 4:**
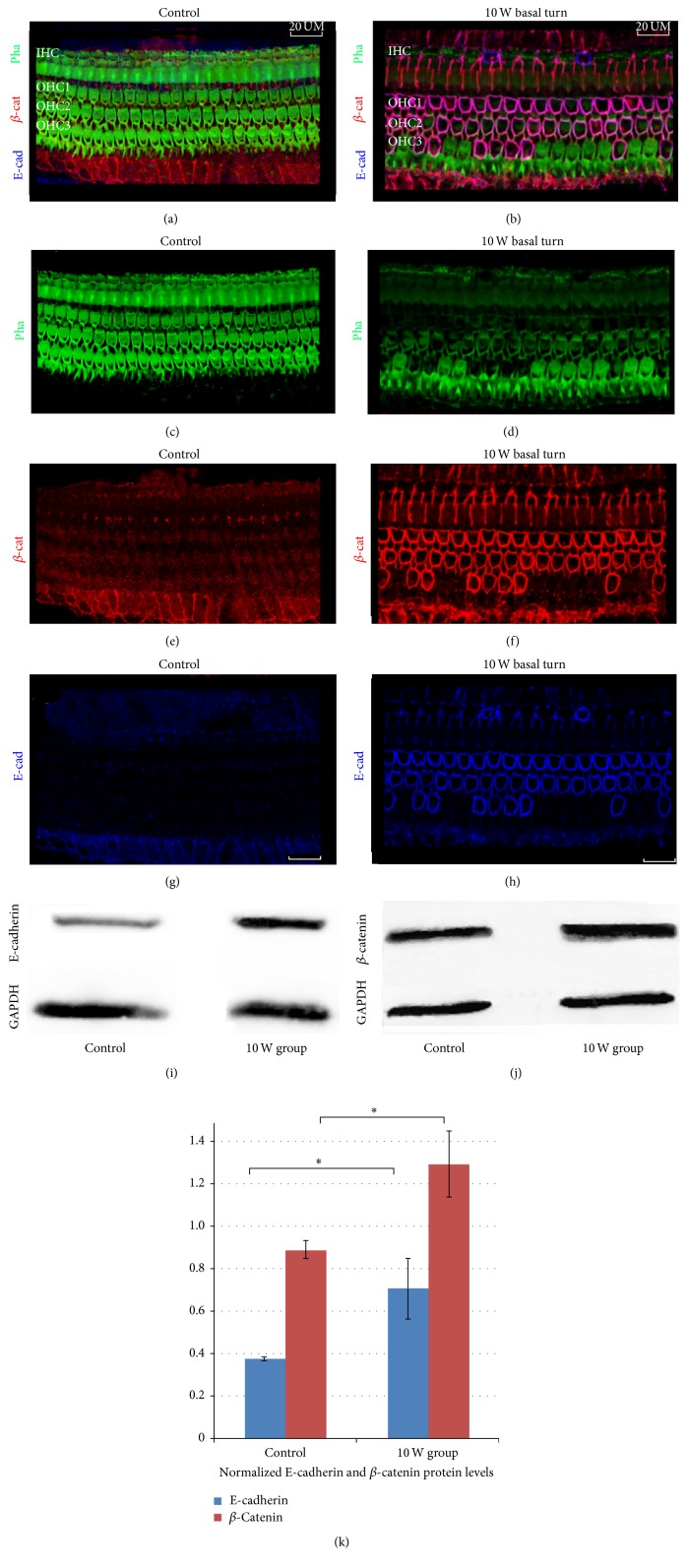
The expression of *β*-catenin and E-cadherin in the sensory epithelium changed after applying CO_2_ laser irradiation. Surface views of the organ of Corti in the basal turn ((a), (c), (e), and (g)) in the control group and ((b), (d), (f), and (h)) in the 10 W laser group. The samples were stained for phalloidin (green, (c), (d)), *β*-catenin (red, (e), (f)), and E-cadherin (blue, (g), (h)). In the 10 W laser group, we observed overexpression of *β*-catenin (red) and E-cadherin (blue) in the damaged surface of the outer hair cells. ((i), (j)) Western blot analysis of E-cadherin (i) and *β*-catenin (j) proteins in the cochleae of the 10 W laser and control groups, which is quantified and plotted in (k). The densities of the E-cadherin and *β*-catenin protein bands were normalised with GAPDH ((i), (j)). Data are presented as means + SD; *n* = 15, ^*∗*^ *P* < 0.05. Scale bar: 20 *μ*m. pha: phalloidin; E-cad: E-cadherin; *β*-cat: *β*-catenin. Scale bar: 20 *μ*m.

The basilar membranes of basal turn on immunofluorescent staining showed positive E-cadherin and *β*-catenin, corresponding to cell-cell adhesion, after the application of a 10 W CO_2_ laser. Images of the basal turn from the 10 W CO_2_ laser group are shown in Figure 4. In the control group (Figures 4(a), 4(c), 4(e), and 4(g)), the inner and outer hair cells expressed almost no *β*-catenin (red, (e)) or E-cadherin (blue, (g)). However, in the 10 W laser group (Figures 4(b), 4(d), 4(f), and 4(h)), we observed strong expression of *β*-catenin (red) and E-cadherin (blue) in the damaged surface of the outer hair cells. The collapsed outer hair cell showed faint phalloidin (green, Figure 4(d)) and bold *β*-catenin (red, Figure 4(f)) and E-cadherin (blue) (Figure 4(h)). Western blot analysis of the proteins E-cadherin (Figure 4(i)) and *β*-catenin (Figure 4(j)) in the 10 W laser and control groups was quantified and is provided in Figure 4(k). The densities of the E-cadherin and *β*-catenin protein bands were normalised with housekeeping proteins GAPDH. The levels of E-cadherin (0.71 ± 0.14) and *β*-catenin (1.29 ± 0.16) were found to be significantly increased in CO_2_ laser injuries (the 10 W laser group) compared with the control group (0.37 ± 0.01 and 0.89 ± 0.05, respectively; *P* < 0.05).

## 4. Discussion

The CO_2_ laser used in this research has a handheld energy delivery system with a hollow-core fibre that can transfer the laser beam at various angles and distances and comes with various handheld accessories of differing lengths and flexibility. These devices expand the application of CO_2_ lasers in ENT surgery [10–12]; fibre-enabled CO_2_ has practical advantages, especially in cases with complex anatomical conditions [12]. In the guinea pig, a temporal bone study had already indicated that laser settings of between 4 and 10 W were sufficient for creating the cochleostomy [7]. In this study, we used a 10 W fibre-enabled CO_2_ laser to establish an inner ear injury with hearing loss and damaged ultrastructure.

In our study, the 10 W CO_2_ laser (606 J/cm^2^) used for cochleostomy produced a rise in temperature of 8.92 ± 2.13°C in the membrane of the round window. Kamalski et al. also observed such thermal effects when evaluating temperature increases during exposure to laser irradiation [8]. Along with an increase in temperature, the ABR threshold also increased by 47.5 ± 10.9 dB SPL in our 10 W CO_2_ laser group. These findings demonstrated a strong relationship with the morphological changes observed within the cochleae. The ultrastructure of the basal turn of the cochlea in laser-treated animals showed various changes, whereas no obvious ultrastructure change was found in the rest parts of the cochlea. Changes in the outer hair cells were more evident than in the inner hair cells, particularly in the third row as seen in our SEM examinations. Most of the outer hair cells had collapsed, and derangements and even loss of the stereocilia were observed, while the outer hair cells acted as cochlear amplifier. Prestin on the outer hair cell membrane is the basis of cochlear amplification in mammals [13]. Okunade and Santos-Sacchi found that prestin is remarkably responsive to fast temperature jumps [14]. We assume the alteration of the prestin may partly explain the irreversible hearing loss.

The E-cadherin/*β*-catenin complex plays an important role in maintaining epithelial integrity, and disrupting this complex affects not only the adhesive repertoire of a cell but also the Wnt-signalling pathway [15]. However, the question of whether and how the Wnt-signalling pathway plays a role in thermal injuries of the inner ear following laser application remains unanswered. In our experiment, we observed that the areas of injured hair cells showed positive E-cadherin and *β*-catenin expressions after excessive laser irradiation. Furthermore, there were strong colocalised expressions of E-cadherin and *β*-catenin in the membrane of the injured hair cells and around the supporting cells. Karpowicz et al. showed that the overexpression of E-cadherin can aid the self-renewal of neural stem cells and increases their number in vitro [16]. In addition, the research of Chen et al. suggested that the overexpression of E-cadherin by viral transduction was sufficient to enhance the generation of induced pluripotent stem cells [17]. That is to say, the upregulation of E-cadherin in the membrane may suggest stronger cell-cell adhesions in the surfaces of the injured hair cells to protect the hair cells from further damage or to enhance the self-rehabilitation of the injured cells after laser irradiation.

In our experiment, there was increased expression of *β*-catenin in the 10 W laser group, particularly in the membrane. We therefore assumed that *β*-catenin was redistributed on behalf of a rise in cell-cell adhesion following hair cell damage, and this may protect the hair cells from further damage by moving from the cytoplasm and nucleus to the membrane and activating the Wnt pathway synergistically with E-cadherin. It may be useful to explore the assumption that the Wnt pathway could lead to regeneration under some conditions, such as after damage. How *β*-catenin-related canonical Wnt signalling influences the rehabilitation or regeneration of damaged hair cells after thermal injury has not yet been demonstrated. Therefore, further studies are required in the future.

Results have shown that when the CO_2_ laser exceeds the safe operating levels, this results in hair cell collapse and deranged stereocilia, particularly in the outer hair cells and, more surprisingly, causes the upregulation of E-cadherin and *β*-catenin in the injured hair cell membranes, suggesting that these could play a role in the overall injury mechanism. Stimulation of Wnt/*β*-catenin may be an avenue to explore for the replacement of adult cochlear hair cells, a sought-after goal for the treatment of sensorineural deafness, which is commonly caused by the loss of hair cells in humans.

## Figures and Tables

**Table 1:** Applied CO_2_ laser mode and parameters.

Laser mode	Power	Spot diameter	Pulse duration/ interval	Working distances	Energy/impulse	Energy density	Number of pulses
SuperPulse	10 W	458 *μ*m	100 ms/100 ms	3 mm	1 J	606 J/cm^2^	2

W: watt; J: joule.

## References

[1] Perkins R. C. (1980). Laser stapedotomy for otosclerosis. *Laryngoscope*.

[2] Lesinski S. G., Palmer A. (1989). Lasers for otosclerosis: CO2 vs. Argon and KTP-532. *Laryngoscope*.

[3] Jovanovic S., Schönfeld U., Fischer R. (1998). Thermic effects in the "vestibule" during laser stapedotomy with pulsed laser systems. *Lasers in Surgery and Medicine*.

[4] Kiefer J., Tillein J., Ye Q., Klinke R., Gstoettner W. (2004). Application of carbon dioxide and erbium:yttrium-aluminum-garnet lasers in inner ear surgery: an experimental study. *Otology & Neurotology*.

[5] Ren D., Sun J., Wan G., Yang F., Shen F. (2005). Influence of carbon dioxide laser irradiation on the morphology and function of guinea pig cochlea. *Journal of Laryngology and Otology*.

[6] Ren D.-D., Chi F.-L. (2008). Experimental study on thermic effects, morphology and function of guinea pig cochlea: a comparison between the erbium:yttrium-aluminum-garnet laser and carbon dioxide laser. *Lasers in Surgery and Medicine*.

[7] Fishman A. J., Moreno L. E., Rivera A., Richter C.-P. (2010). CO_2_ laser fiber soft cochleostomy: development of a technique using human temporal bones and a guinea pig model. *Lasers in Surgery and Medicine*.

[8] Kamalski D. M. A., Verdaasdonk R. M., De Boorder T., Vincent R., Trabelzini F., Grolman W. (2014). Comparison of KTP, Thulium, and CO_2_ laser in stapedotomy using specialized visualization techniques: thermal effects. *European Archives of Oto-Rhino-Laryngology*.

[9] Luo W.-W., Yang J.-M., Han Z. (2014). Atoh1 expression levels define the fate of rat cochlear nonsensory epithelial cells in vitro. *Molecular Medicine Reports*.

[10] Albers A. E., Wagner W., Stölzel K., Schönfeld U., Jovanovic S. (2011). Laser stapedotomy. *HNO*.

[11] Remacle M., Ricci-Maccarini A., Matar N. (2012). Reliability and efficacy of a new CO_2_ laser hollow fiber: a prospective study of 39 patients. *European Archives of Oto-Rhino-Laryngology*.

[12] Brase C., Schwitulla J., Künzel J., Meusel T., Iro H., Hornung J. (2013). First experience with the fiber-enabled CO2 laser in stapes surgery and a comparison with the “one-shot” technique. *Otology and Neurotology*.

[13] Song L., Santos-Sacchi J. (2010). Conformational state-dependent anion binding in prestin: evidence for allosteric modulation. *Biophysical Journal*.

[14] Okunade O., Santos-Sacchi J. (2013). IR laser-induced perturbations of the voltage-dependent solute carrier protein SLC26a5. *Biophysical Journal*.

[15] Tian X., Liu Z., Niu B. (2011). E-Cadherin/*β*-catenin complex and the epithelial barrier. *Journal of Biomedicine and Biotechnology*.

[16] Karpowicz P., Willaime-Morawek S., Balenci L., Deveale B., Inoue T., Van Der Kooy D. (2009). E-Cadherin regulates neural stem cell self-renewal. *Journal of Neuroscience*.

[17] Chen T., Yuan D., Wei B. (2010). E-cadherin-mediated cell-cell contact is critical for induced pluripotent stem cell generation. *Stem Cells*.

